# Qualitative Study of Barriers to Adherence to Antihypertensive Medication among Rural Women in India

**DOI:** 10.1155/2019/5749648

**Published:** 2019-01-23

**Authors:** Shreya Gupta, Jas Pal Dhamija, Indu Mohan, Rajeev Gupta

**Affiliations:** ^1^Intern, Department of Medicine, Mahatma Gandhi Medical College, Jaipur, India; ^2^Associate Professor, Department of Medicine, Mahatma Gandhi Medical College, Jaipur, India; ^3^Assistant Professor, Academic & Research Development Unit, Rajasthan University of Health Sciences, Jaipur, India; ^4^Chair, Academic & Research Development Unit, Rajasthan University of Health Sciences, Jaipur, India; ^5^Department of Preventive Cardiology & Internal Medicine, Eternal Heart Care Centre and Research Institute, Jaipur, India

## Abstract

**Objective:**

There is poor adherence to antihypertensive therapies among women in India. To determine its socioeconomic barriers we performed a qualitative study on Indian rural women with hypertension.

**Methods:**

In-depth interviews with women having hypertension and presenting to outpatient department at a teaching hospital were performed in 30 women aged 35-65 years, using a questionnaire focused on reasons for nonadherence and poor lifestyle modification. Low to medium adherence was observed in two-thirds of women.

**Results:**

Majority of women were from low socioeconomic status and were illiterate. Awareness of hypertension and its complications was poor. Knowledge and practices of cessation of smoking and tobacco use and salt restriction in hypertension were low. Efforts to increase physical activity and decrease dietary fat and sugar intake were largely absent. Local follow-up at rural community health centres was not practiced due to physician nonavailability and about half used alternative systems of medicine. None had health insurance or access to free medicines. All the women had to pay out-of-pocket for medicines and were concerned with cost of therapy as well as pill burden. Half of the women borrowed money from relatives or friends to reach the hospital and pay for medicines.

**Conclusions:**

Socioeconomic barriers for low adherence to antihypertensive medication in women in India are low awareness of hypertension and complications, poor access to care, out-of-pocket payments, borrowing money, lack of insurance, and cost of medicines.

## 1. Introduction

Hypertension is a widely prevalent disorder and affects more than a billion individuals worldwide [1]. Global Burden of Diseases Study has reported that high blood pressure (BP) is the most important risk factor for mortality as well as disease burden and is responsible for 15-18% of annual deaths [2]. High prevalence of hypertension is reported from various regions of India [3]. Recent studies have reported that hypertension is present in 25-30% urban and 10-15% rural subjects in India [4–6]. This extrapolates to more than 200 million patients with hypertension in the country [6]. An important metric absent from these estimates is the prevalence of uncontrolled hypertension. Studies from Indian report that uncontrolled or poorly controlled hypertension is widely prevalent [3]. In developed countries it has been reported that 30-40% patients with hypertension are not controlled to target while this proportion in low and lower-middle income developing countries is 60-70% in urban and 80-90% in rural populations [7]. It has been reported that properly controlled BP in patients with hypertension can reduce cardiovascular mortality by as much as 30% [8].

Multiple factors are responsible for poor BP control in developing countries [9, 10]. These vary from macrolevel health system and healthcare provider factors to individual patient level factors [10–13]. Most of these factors have been well studied in developed countries. There have been limited studies that have qualitatively determined reasons for low hypertension awareness, treatment, and control in India [14–16] or other low and lower-middle income countries [17–21]. The health system factors that have been found important are rural residence and nonavailability of healthcare facilities. Individual factors are poverty, low educational status, low spousal education, cost of drugs and healthcare, personal factors (psychosocial, health-related inertia), and drug-related issues. Therefore, to evaluate various social and economic factors that are considered as barriers to hypertension treatment and control, we designed a qualitative study on rural women with hypertension presenting to our hospital. This qualitative study evaluated barriers and other determinants of hypertension awareness, treatment, and control among rural women attending outpatient clinic at a medical college hospital in Jaipur, India.

## 2. Methods

The study was funded under the Short Term Studentship Program of Indian Council of Medical Research, Government of India, and approved by the institutional ethics committee of the medical college. Informed consent to participate was obtained from all women.

Successively presenting rural women with diagnosed hypertension in the department of medicine outpatient-unit were evaluated for eligibility. Inclusion criteria were as follows: rural women, age 18-70 years, on drug therapy for at least 12 months, and providing informed consent. Patients who did not agree to the informed consent, those having pregnancy or lactation, and those with a recent acute coronary event, stroke, or any critical medical illness or having multiple comorbidities were excluded. Demographic details (weight, height, body mass index, BP measurements) and prescription details were obtained. A questionnaire was used to assess the level of adherence to antihypertensive medications. This was followed by asking open-ended questions using a semistructured questionnaire followed by a free conversation.  Table 1 shows the summary of the semistructured questionnaire and the free conversation. This has been adapted from study by Legido-Quigley et al. [19]. A Hindi version of this questionnaire was used and administered by the medical student (SG).

*Statistical Analyses*. All the responses were recorded on case report forms. Descriptive statistics are reported.

## 3. Quantitative Results

We interviewed 30 rural women with confirmed hypertension. Sociodemographic and clinical details are reported in Table 2. Mean age was 56+9 years (range 25-69 yr). Majority of women were from low socioeconomic status households, illiterate, and housewives. More than half of the women were physically inactive and prevalence of smoking was low. Lifestyle modifications were advised in a majority of patients and most were aware of the importance of stopping smoking or tobacco use: 4 of 5 women advised on tobacco cessation tried to do so and 6 tried weight loss. On the other hand, salt restriction was practiced in only 4 women. Effort to increase physical activity was in 16, to decrease dietary fat in 26, and to lower sugar intake in 20 women.

Sociodemographic factors and knowledge about hypertension are shown in Table 3. Awareness of hypertension and its risk factors was adequate in only 3, and 16 had some information, while 10 had no knowledge of hypertension or its complications. In this study 27 women had their BP measured within the last 6 months, while in 3 women it was more than 6 months. Although all these patients had access to a medical college hospital only, 12 (36.7%) had visited a local community health centre and 8 (26.7%) had used alternative systems of medicine for treatment in the last 12 months. 56.7% used public transport system for travel to hospital. All the patients had to pay for medicines out-of-pocket. Only 2 women (6.7%) had some sort of insurance or social security. A large proportion of women, 15 women (50.0%), borrowed money from relatives or friends to reach the hospital and pay for medicines. The most commonly prescribed drugs were amlodipine (n=14) and telmisartan (n=13). Angiotensin receptor blockers (the most expensive) were prescribed in 23 patients and calcium channel blockers in 16 while low-cost drugs such as diuretics (n=7) and beta blockers (n=3) were prescribed in low proportions. Combination therapies comprising 2 or 3 drugs were prescribed in 13 women (43.3%).

## 4. Qualitative Results

We explored knowledge, behavior, practices, and healthcare experiences of rural women with hypertension in relation to its prevention and treatment. Low adherence to medicines and healthy lifestyles were observed among this cohort of women. Reasons for these include low awareness of complications of hypertension, poor access to care, out-of-pocket payments, borrowing money, lack of insurance, prescriptions of more expensive medicines, and low use of combination pharmacotherapy. Previous studies have reported that age is an important factor which influences adherence and older patients are better adherent compared to the younger [11]. Previous studies also reported better health awareness and knowledge of high blood pressure and its complications in older patients. We have similar observations. It has been shown that improving patients' knowledge (health literacy), whether of the illness or of the medications prescribed, results in better adherence to medications. Getting patients involved in their treatments by imparting relevant knowledge often empowers patients to be more concerned about their health. In our study almost all participants felt that they were adherent to prescribed medications. However, on direct assessment only a third were found to have high level of adherence to their antihypertensive medication. These findings are similar to studies from Mangalore (India) and Malaysia where the good and medium adherence were 69.4% and 53.4%, respectively [16, 20].

The most common cause for missing a dose was patient's forgetfulness. Statements such as “I do not have time to take medicine”; “I have lot of other things to do”; “Doctor advised me to take medicine in morning and when I forget, I take it the next day”; “I feel sick if I have to take medicines” abound. This shows that all possible means should be taken to enhance patient's memory; healthcare professionals should counsel to help patients organize this aspect. Significant improvement is possible in primary health clinics by training and mobilizing the healthcare providers [20]. We focused on the following four key themes, adapted from Legido-Quigley et al. [19].

*(a) Patient's Experiences*. A few patients were asymptomatic but were found to have high blood pressure during routine medical examinations. Headaches, dizzy spells, feeling faint, etc. were some of the acute symptoms experienced by some patients whereas others experienced insidious symptoms such as feeling generally unwell, tired, or depressed and nervous. Awareness of the causes of hypertension was scarce and none could recall attending any campaigns to increase knowledge about it. Risk factors such as high fat diet, high salt intake, and less exercise were commonly observed in their lifestyles. Majority of the interviewees were unaware of the asymptomatic nature of hypertension and the rationale for its treatment. These findings are similar to studies in Colombia and Malaysia [19, 20].

*(b) Patients' Attitudes*. Nonpharmaceutical management of hypertension can be achieved by regulating the dietary patterns and maintaining exercise routines [22]. Most of the interviewees were vegetarian, consuming wheat, rice, maize, pulses, fried food, and sweets, with variance seen in the variety and quantity according to their income. Healthy diet comprising fruits and vegetables was unaffordable. This is similar to a recent study in a low income country [21]. Smoking was present in only a few. All the patients were advised to change their diet by their doctors. Most of the patients had reduced their salt intake to a great extent or completely eliminated added salt from their diet after being advised of the same. There was a great reduction seen with fried food and patients were including more salads, vegetables, and fruits in their diets, as a result of which several patients had lost weight. Issues relating to availability and affordability of healthy food options still persist. Another important lifestyle change for participants was greater exercise. They had been advised to walk every day and the majority reported an effort to do so. Participants who were aged and had other problems such as arthritis found it difficult to implement. Several interviewees reported walking daily for 20-30 minutes.

*(c) Health System Barriers*. People who lived far away from the hospital preferring to avoid journey and paying the transportation costs because of low income were found to skip doses of their medication regime. Those who went to the hospital after paying the necessary transportation costs find that sometimes the medicines are not in stock in the hospital, ultimately ended up buying them from the pharmacy which proves costlier to them. The problem seemed especially great when medicines were expensive. Cost as barrier to therapies for cardiovascular diseases in India and other low income countries has been reported earlier [12]. Table 3 shows that the most commonly prescribed drugs were expensive angiotensin receptor blockers (e.g., telmisartan). Financial help from family members and neighbors helped overcome the problem in some cases. Partners help each other by reminding each other to take their medication; daughters-in-law help their parents by accompanying them to the doctor, keeping a track of their medication, and contributing financially. Describing the lack of support the interviewees recounted experiencing stress and difficulties accessing services.

*(d) Healthcare Professionals*. The relationship with the doctor emerged as the most important factor for adherence to medication. Some professionals are praised for being empathetic, communicative, and pleasant while others are criticized for being distant, uncaring, and not providing sufficient information. There was an overwhelming communication problem with interviewees reporting that they did not share information with their doctors or ask questions when they had doubts regarding the treatment. Limited time available for each consultation contributes to the problem. Interviewees typically reported that their doctors did not explain what it means to have high blood pressure or how it can be prevented. A trusting relationship was difficult to establish as the interviewees reported seeing different doctors each time. Fear of giving information to health workers was also reported.

## 5. Discussion

This study shows that barriers to hypertension control in Indian rural women range from issues related to lack of information, a low priority given to hypertension in comparison to their other health problems, economic constraints in accessing medication and facilities, and distrusting the healthcare system and the quality of the care provided in public healthcare settings. Interviewees highlighted several areas that need improvement. Most referred to the need for quicker access to healthcare and receiving good quality medications for free. There have been calls for more easily available healthcare facilities and specialist care in India [23]. The present study shows that the problems of access and costs are high in rural areas of the country.

There are multiple limitations of the present study. A qualitative study is useful in assessment of an individual patient's role in monitoring, maintenance, and management of chronic diseases [24]. The data for such studies could be obtained for evaluation of multiple theories regarding differences that influence adherence to lifestyles and therapies: ethnographic, phenomenological, or grounded theory [25]. We proposed the latter at it is grounded in sociological realities and real world experiences [26, 27]. Data could be obtained by either one-to-one interviews (which we used) or focused-group discussions, ethnomethodology, auto-ethnomethodology, and discursive analyses [28, 29]. Other methods to obtain data and perform analyses are conversation analysis, critical social theory, feminist research, hermeneutics, historical research, narrative research, participatory action research, community level participatory research, and content analysis [25]. We used one-to-one interview which is more useful in a chronic self-managed condition such as hypertension and used the method of content analysis. Multiple barriers were identified as reported above. Focused group discussions in the present context could also be used to identify health system related and community related barriers [30], and this is a study limitation. We used a purposive sampling methodology, which is the most used strategy in a qualitative study [28], especially when the focus is on specific questions, as in our case—nonadherence to medications. Random sampling method is more useful in a quantitative study when the scope of the problem is the study question.

Small sample size from a single centre and defined geographical distribution may be considered a study limitation but most qualitative studies are performed using small sample sizes. There are multiple methods of data analyses of qualitative studies: content analysis, thematic analysis, and grounded-theory analysis reported above [28]. We used content analyses (simple enumeration) but did not use the computerized methods of analyses due to nonavailability of this technology at our centre. However, most of the studies use the methods used in the present study. In the present study we have interviewed for barriers to adherence and not its facilitators and this is also a study limitation. Multiple psychosocial theories exist to explain poor adherence to chronic disease therapies. Content analysis in the present study shows a mixture of low self-esteem, entrenched beliefs, preconceived notions, lack of true knowledge, and negative affect and emotions as important factors. Larger studies at multiple sites in the country are needed to assess local-level barriers to adherence using qualitative study design [31]. Also required are intervention studies to evaluate influence of behavior change to promote adherence to hypertension related lifestyles and medications to develop generalizable results. The present study, thus, highlights the importance of poor patient access, health education and empowerment, and financial support at patient level and greater use of low-cost generics and combination therapies by the physicians. A systemwide change in healthcare delivery in India [21] is needed to control the epidemic of hypertension related diseases in India and other low and lower-middle income countries.

## Figures and Tables

**Table 1:** Summary interview guide.

**KNOWLEDGE AND DIAGNOSIS**

(i) Can you tell us about your first symptoms of high BP? Do you have other health problems?

(ii) How did you decide to seek care? Did the family help in this process?

(iii) How much do you know about high BP?

(iv) To what extent do you think high BP is an important disease?

**TREATMENT**

(i) What was the treatment that was first prescribed? Was it subsequently changed? In what way?

(ii) What is your usual mode of payment? (Health insurance plan/current income/sold items/borrowed, etc.)

(iii) If any, what difficulties did you face during this process? (related to health system, family, work, etc.)

(iv) Have you received an advice on preventive measures and lifestyle changes? Have you followed it?

(v) Did you have any side effects because of your medication?

(vi) When was the last time you had your BP measured? What prevents you from measuring it regularly?

(vii) Have you ever had any cardiac tests?

**ACCESS AND USE**

(i) Are the drugs and consumables available? Or access problems to the facilities? Discuss problems.

(ii) How far is your usual healthcare facility?

(iii) What kind of health insurance do you have and what does it cover?

(iv) Have you got a particular doctor or health professional who is mainly looking after you and knows you well?

**Healthcare Experience and Recommendations**

(i) How would you assess your communication with health providers you have encountered?

(ii) To what extent have you been kept informed about your treatment?

(iii) Have you heard of any initiative to improve prevention of HT?

(iv) Are there any changes that need to be made outside the healthcare system?

**Table 2:** Sociodemographic characteristics and risk factors.

**Variables (n=30)**	**Prevalence (**%**)**
Age	56.5+8.9 (range 25-69)

Educational status	
Illiterate/primary	21(70.0)
Secondary	9 (30.0)

Occupation	
Housewife	22 (73.3)
Manual labor	6 (20.0)
Others	2 (6.7)

Income (Indian rupees INR) (1 Euro=80 INR)	
<15,000 per year	14 (46.7)
16,000-30,000 per year	10 (33.3)
>30,000 per year	6 (20.0)

Smoking/tobacco use	5 (16.7)

Physical activity patterns	
Physical inactivity	16 (53.3)
Leisure-time physical activity	4 (13.3)

Attempts at lifestyle change advised/modified	
Smoking/tobacco use	5/4 (80.0)
Physical inactivity	29/16 (55.1)
Weight reduction	6/21 (28.6)
Dietary salt control	30/4 (13.3)
Greater fruit/vegetable intake	30/5 (16.7)
Reduction of fried foods	29/26 (89.6)
Reduction of sugar sweetened beverages	28/20 (71.4)

**Table 3:** Hypertension-specific behavioral factors.

**Variables (n=30)**	**Prevalence (**%**)**
Adherence to drug treatment	
High	10 (33.3)
Medium	9 (30.0)
Low	11 (36.7)

Knowledge of hypertension	
No knowledge	10 (33.3)
Little knowledge	16 (53.3)
Familiar	3 (10.0)

BP measurements	
Within 1 month	0
Within 6 months	27 (90.0)
> 6 months ago	3 (10.0)

Transport to hospital	
Public transport	19 (63.3)
Private car/motorbike	6 (20.0)
Walk	5 (16.7)

Mode of payment of fees/medicines	
Current income/savings	30 (100.0)
Borrowing	15 (50.0)
Mortgage	1 (3.3)
Health insurance	2 (6.7)

Healthcare utilization in past 12 months	
Community health centre	11 (36.7)
Alternative medicine specialist	8 (26.7)
Emergency room visit	2 (6.7)

Anti-hypertensive drugs prescribed	
Angiotensin receptor blockers	23 (76.7)
Calcium channel blockers	16 (53.3)
Thiazide-type diuretics	7 (23.3)
Beta blockers	3 (10.0)
Centrally acting alpha blockers	1 (3.3)
Aldosterone antagonist	1 (3.3)

Drug prescribing patterns by physicians	
Single drug	17 (56.7)
Drug combinations	13 (43.3)

## Data Availability

There are no data available beyond that included in this manuscript.
